# How much rotational error is clinically acceptable for single‐isocenter/two‐lesion lung SBRT treatment on halcyon ring delivery system (RDS)?

**DOI:** 10.1002/acm2.14068

**Published:** 2023-06-13

**Authors:** Damodar Pokhrel, Richard Mallory, Mark E. Bernard, Mahesh Kudrimoti

**Affiliations:** ^1^ Department of Radiation Medicine University of Kentucky Lexington Kentucky USA

**Keywords:** Halcyon RDS, patient set up errors, SIMT, synchronous Lung SBRT, target coverage loss

## Abstract

**Purpose:**

SBRT treatment of two separate lung lesions via single‐isocenter/multi‐target (SIMT) plan on Halcyon RDS could improve patient comfort, compliance, patient throughput, and clinic efficiency. However, aligning two separate lung lesions synchronously via a single pre‐treatment CBCT scan on Halcyon can be difficult due to rotational patient setup errors. Thus, to quantify the dosimetric impact, we simulated loss of target(s) coverage due to small, yet clinically observable rotational patient setup errors on Halcyon for SIMT treatments.

**Methods:**

Seventeen previously treated 4D‐CT based SIMT lung SBRT patients with two separate lesions (total 34 lesions, 50 Gy in five fractions to each lesion) on TrueBeam (6MV‐FFF) were re‐planned on Halcyon (6MV‐FFF) using a similar arc geometry (except couch rotation), dose engine (AcurosXB algorithm), and treatment planning objectives. Rotational patient setup errors of [± 0.5⁰ to ± 3.0⁰] on Halcyon were simulated via Velocity registration software in all three rotation axes and recalculated dose distributions in Eclipse treatment planning system. Dosimetric impact of rotational errors was evaluated for target coverage and organs at risk (OAR).

**Results:**

Average PTV volume and distance to isocenter were 23.7 cc and 6.1 cm. Average change in Paddick's conformity indexes were less than −5%, −10%, and −15% for 1°, 2°, and 3°, respectively for yaw, roll, and pitch rotation directions. Maximum drop off of PTV(D100%) coverage for 2° rotation was −2.0% (yaw), −2.2% (roll), and −2.5% (pitch). With ±1° rotational error, no PTV(D100%) loss was found. Due to anatomical complexity: irregular and highly variable tumor sizes and locations, highly heterogenous dose distribution, and steep dose gradient, no trend for loss of target(s) coverage as a function of distance to isocenter and PTV size was found. Change in maximum dose to OAR were acceptable per NRG‐BR001 within ±1.0° rotation, but were up to 5 Gy higher to heart with 2° in the pitch rotation axis.

**Conclusion:**

Our clinically realistic simulation results show that rotational patient setup errors up to 1.0° in any rotation axis could be acceptable for selected two separate lung lesions SBRT patients on Halcyon. Multivariable data analysis in large cohort is ongoing to fully characterize Halcyon RDS for synchronous SIMT lung SBRT.

## 1 INTRODUCTION

Lung metastases frequently present with multiple lesions, causing extensive complications during treatment planning and delivery. Various studies have demonstrated the efficacy of treating multiple primary or metastatic lung cancer patients using stereotactic body radiation therapy (SBRT).^1^, ^2^, ^3^, ^4^, ^5^ Traditionally, SBRT treatment of multiple lung metastases involves treating each lesion consecutively, placing one isocenter per target. However, each isocenter requires taking a CBCT image and applying a shift which leads to extensive treatment times. The patient, often times elderly, is required to remain perfectly still for the entirety of the treatment. This demand is unrealistic and may result in significant discomfort in addition to the symptoms of their lung cancer. Our clinical experience indicates these patients are not likely to tolerate a long treatment time and will often shift from the treatment position, causing intrafraction motion error. In an effort to increase the patient comfort, compliance, and clinical efficiency, many centers have begun treating oligometastatic lung patients via a single‐isocenter/multi‐target SBRT (SIMT‐SBRT) technique which has been shown to be similar in plan quality and significantly improve treatment delivery efficiency compared to a multi‐isocenter approach.^4^, ^5^, ^6^, ^7^, ^8^, ^9^ This can decrease treatment time significantly, improve patient convenience and clinical efficiency, thereby reducing the chances of patient movement during treatment or being unable to tolerate these complex SBRT treatments as reported by Pokhrel et al. in a prospective clinical follow up study.^9^

One of the major challenges of image‐guided SIMT to multiple lung lesions is that lung tumor could move independently to each other with the breathing motion. Another difficulty arises when utilizing this SIMT approach is the potential increase of dosimetric error, particularly due to rotational patient setup errors as reported by many researchers.^10^, ^11^, ^12^, ^13^, ^14^, ^15^ Typically, this is combated by utilizing a six degrees‐of‐freedom (6DOF) couch that applies rotational and translational shifts obtained from the pre‐treatment CBCT taken for set‐up verification.^10^, ^15^ Recently, Gao et al provided a quality assurance (QA) procedure for maintaining the clinical standard of off‐axis Winston‐Lutz QA for SIMT stereotactic treatment.^16^ However, 6DOF couches are typically only available in highly advanced and specialized machines that are cost prohibitive for smaller clinics to obtain and maintain on a regular basis. Therefore, there is a need for the characterization of worst‐case scenarios for the dosimetric impact of the patient rotational setup errors in the SIMT setting. This will be followed by methods to correct the patient rotational setup errors that would be applicable to ring delivery systems (RDS) such as Halcyon linacs (Varian Medical Systems, Palo Alto, CA).^17^ Thus, the availability of SIMT treatment option and it is characterization on Halcyon RDS could be beneficial to the rural community cancer centers, allowing more access of high‐quality therapeutic radiation treatments for an underserved patient cohort.

The Halcyon RDS has a dual‐layered staggered multileaf collimator (MLC) design that eliminates the need for jaws in favor of ultra‐low leakage and transmission through the MLCs with a virtual MLC resolution of 5 mm at isocenter, similar to the standard Millennium 120 MLC found on the standard TrueBeam linac. The Halcyon gantry can rotate up to four times the speed of conventional C‐arm linacs and has double the MLC speed (maximum up to 5 cm/s) for it's single‐energy 6MV‐FFF beam.^17^, ^18^, ^19^, ^20^, ^21^, ^22^ The high speed and ultra‐low leakage of MLCs would theoretically reduce the dose bridging in between the lesions for a dual‐target single‐isocenter setting compared to the aforementioned Millennium 120 MLC.^20^ Additionally, the Halcyon streamlines patient setup procedure via an optimized one‐step patient set‐up, improved CBCT image quality and imaging time (15–30 s for one CBCT scan), and auto‐registration at the treatment console which in turn reduces total treatment time for VMAT SBRT cases, significantly improving patient comfort and clinic efficiency.^23^, ^24^ As reported by previous researchers, Halcyon has been shown to be theoretically capable of delivering dosimetrically similar quality plans to TrueBeam for SIMT‐SBRT treatments.^28^, ^29^, ^30^, ^31^, ^32^ This, in tandem with its 6MV‐FFF beam with a maximum available dose rate of 800 MU/min and proven mechanical stability of the off‐axis treatment fields makes it a promising candidate for complex treatment including SIMT‐SBRT^28^, ^29^, ^30^, ^31^, ^32^ following AAPM SBRT/SRS protocols guidelines.^25^, ^26^, ^27^, ^33^, ^34^

However, the current Halcyon RDS does not have any rotational setup correction capability, owing to its simplified design and ring gantry bore that does not allow for couch rotation.^17^ Thus, quantifying and characterizing the dosimetric impact of the worst‐case scenario of patient setup errors, assuming interfractional errors are systematic and consecutive, allows for understanding the limits of acceptable operation or the necessity for alternative rotational correction methods for Halcyon RDS in the SIMT‐SBRT setting. In turn, this could allow for increased utilization of the Halcyon in single linac clinics. This could ease clinic workflow by selectively treating patients in the large academic centers with high SBRT patient volume, and allow managing oligometastatic SBRT treatments in the smaller rural clinics with only a Halcyon linac. In addition, this work could provide tremendous support for patient selection, treatment planning and delivery of SBRT treatments to underserved patient population in cancer centers in 3rd world countries with less physics support.

## 2 MATERIALS AND METHODS

After obtaining Institutional Review Board approval from our institution, 17 patients (eight bilateral and nine unilateral lungs) with two synchronous metastatic lung tumors who underwent SIMT lung SBRT treatment on a TrueBeam linac for 50 Gy in five fractions were retrospectively included in this simulation study on Halcyon RDS.

### 2.1 Patient setup and contouring

These patients were immobilized using the Body Pro‐Lok™ SBRT system (CIVCO, Orange City, IA) in the supine position with arms above the head. A free‐breathing helical CT scan was acquired on a SOMATOM.go CT simulator (Malvern, PA) with 512 × 512‐pixel image size and 1.25 mm slice thickness. Respiratory assessment and motion management included abdominal compression and a 4D‐CT scan via Varian RPM system (version 2.7). The 3D CT scan was brought into Eclipse Treatment Planning System (Version 15.6, Varian Medical Systems, Palo Alto, CA). With a 4D‐CT scan, an internal target volume (ITV) was contoured based on the registered 4D‐CT reconstructed maximum intensity projection (MIP) images. The planning target volumes (PTVs) were created by expanding a uniform margin of 5 mm from each ITV. The two targets were labeled arbitrarily as PTV1 and PTV2 by the treating physicians. All planning was completed on the free‐breathing untag CT images and Hounsfield units within the PTV were maintained per the planning CT dataset following our in‐house SBRT protocol. Critical structures were delineated including lungs (right, left and combined), spinal cord, heart/pericardium, trachea and bronchus tree, esophagus, skin and ribs (right, left and combined).

### 2.2 Clinical SIMT lung SBRT plans

**FIGURE 1:**
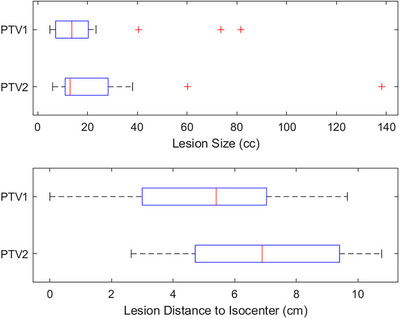
Box and whisker plot demonstrating distribution of variable lesion sizes (top plot) and lesions distance to isocenter (bottom plot) with a total of 34 lesions from 17 SIMT lung SBRT patients simulated on Halcyon RDS.

For all 17 patients, clinical SIMT lung SBRT plans were generated in the Eclipse treatment planning system (TPS) for treatment on a TrueBeam linac v2.7 (Varian Medical Systems, Palo Alto, CA) using the standard millennium 120 multileaf collimators and 6 MV‐FFF (1400 MU/min) beam. A single isocenter was placed approximately equidistant between the two tumors, manually. The distance to isocenter was calculated by finding the coordinates of the PTV geometric center of each lesion and calculating Euclidian distance in the 3D geometry with the isocenter coordinates as a reference point. This provided a total of 34 lesions of varying sizes and distance to isocenters as shown in Figure 1. The average size of the 34 lesions was 23.72 ± 27.49 cc. While the standard deviation is larger than the mean in this metric, this is due to a number of outliers as seen in Figure 1. The average distance to isocenter between all 34 lesions was 6.05 ± 2.76 cm.

Prescription dose was 50 Gy in 5 fractions to each lesion simultaneously. Both PTVs (PTV 1 and PTV 2) were planned with dose prescribed to the 70%−80% isodose line to each lesion equivalently and the plan optimized in such a way that 95% of each PTV received 100% of the prescription dose. The maximum dose to the PTV fell inside the ITVs. Treatment plans were generated utilizing either 2 full coplanar arcs (bilateral lesions) or 3 non‐coplanar partial arcs at ±5–10° couch kick based on patient clearance. Optimal collimator angles and jaw‐tracking option were chosen to minimize the MLC leakage and transmission between each arc on TrueBeam linac. Dose was calculated using the Boltzmann‐transport‐based AcurosXB dose engine for tissue heterogeneity corrections (with 1.25 mm dose calculation grid size) with dose‐to‐medium reporting mode.^35^, ^36^, ^37^ Planning objectives followed RTOG‐0813 and oligometastatic NRG‐BR001/002 protocols guidelines.^1^, ^2^, ^38^ Each of the clinical SIMT‐VMAT lung SBRT plans were delivered every other day to the patient in the clinic following the in‐house imaged‐guided procedure. All patients tolerated SIMT lung SBRT treatment well.

### 2.3 Simulated SIMT lung SBRT plans

First, to benchmark the Halcyon RDS, all TrueBeam single‐isocenter/two‐lesion lung SBRT plans were reoptimized for Halcyon RDS coplanar geometry with 2−4 partial/full arcs using Halcyon's 6MV‐FFF beam with a maximum output rate of 800 MU/min. TrueBeam couch structure was replaced by the mandatory Halcyon couch and the SBRT board was used as TrueBeam clinical plans. For all Halcyon plans, the isocenter location, dose calculation algorithm, calculation grid‐size, convergence mode, and Eclipse PO‐MLC algorithm settings were identical to TrueBeam plans. All Halcyon single‐isocenter/two‐lesion lung SBRT plans were normalized to achieve identical combined target coverage to clinical TrueBeam plans providing similar maximum dose to target and dose to organs‐at‐risk (OAR). All Halcyon plans met NRG‐BR001/002 requirements.

Second, to evaluate rotational patient setup uncertainties, clinically observable setup errors in pitch, yaw, and roll axes were simulated in Eclipse TPS on Halcyon. Evaluation of our previously treated SIMT lung SBRT patient setup errors on pre‐treatment conebeam CT scans (for mean and maximum rotational shifts) on TrueBeam linac for thoracic lesions allowed for determination of clinically representative systematic interfraction rotational setup errors to be within ±3° for yaw, roll and pitch direction. The rotational patient setup errors were defined for patient rotations relative to the isocenter around the left to right (yaw), superior to inferior (roll), and anterior to posterior (pitch) axes per Varian IEC scale. For single‐isocenter VMAT treatment, our current clinical practice is that if errors larger than ±3° in any rotational direction are observed, the treatment will be restarted by resetting the patient and re‐imaging for better alignment to the treated lesions.

Due to the coplanar geometry and the lack of 6DOF couch, demonstrating the loss of target(s) coverage due to clinical rotational setup errors on Halcyon was not readily achievable in the Eclipse TPS. Thus, to address this issue, an in‐house simulation method was developed and integrated into Eclipse TPS via Velocity image‐registration software (Varian Medical Systems, Palo Alto, CA) in order to achieve the desired transformations and re‐compute the dose distributions in the simulated SIMT‐VMAT lung SBRT plans. To reproduce the rotational patient setup errors, this simulation method allowed the boundary conditions to confine the systematically generated patient setup uncertainties in between ±0.5⁰ and ±3.0⁰ in each rotational axis with respect to the single‐isocenter location as a reference point, as described above. For each patient's CT dataset, the rotation was performed around each axis for ±0.5⁰, ±1.0⁰, ±2.0⁰ and ±3.0⁰. This was done by exporting the original SIMT‐SBRT CT images from Eclipse TPS to the Velocity registration software. Two copies of the original CT images were registered to each other. One set, including all structure datasets, was rotated with respect to the original CT images one axis at a time with the center of rotation located at the treatment isocenter. For all patients and all simulated with different rotational shifts, a new CT was sampled from the rotated CT dataset and imported into the Eclipse TPS for each rotational angle and axis of rotation. These rotated and resampled CT image sets were registered back to the original CT images, including the rotated structure sets copied and overlaid, and the SBRT plans were recalculated.

### 2.4 Plan comparison and data analysis

**FIGURE 2:**
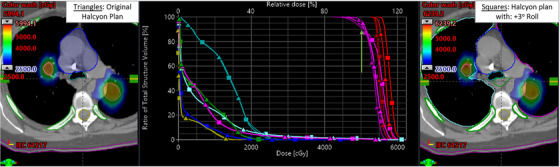
(Example bi‐lateral SIMT Lung SBRT plan on Halcyon, patient #2): Left: Original isodose colorwash distribution of 2‐bilateral lung lesions SIMT‐SBRT plan on Halcyon for 50 Gy in 5 fractions to each lesion, simultaneously. Middle: DVHs parameters, triangles representing the original SIMT plan and squares representing the transformation plan with +3^o^ roll applied to the original plan. Right: corresponding isodose colorwash disribution for the example case. Crosshair shows the isocenter location. The average distance to isocenter was 5.0 cm. In addition to the PCI loss of 11% and 8.5% for PTV1 and PTV2, the vertical green arrow shows the PTV(D100%) loss of both PTV1 and PTV2 with respect to the origial PTVs by 4% and 3% respecitvely. The change in mean dose to ITVs (red) was clinically insignificant, however, significant increase of maximal dose to heart (blue), spinal cord (yellow) and ribs by 9.0 Gy, 2.5 Gy and 3.0 Gy was observed. However, the maximal doses to esophagus/bronchial tree were reduced by 2.3 Gy.

**FIGURE 3:**
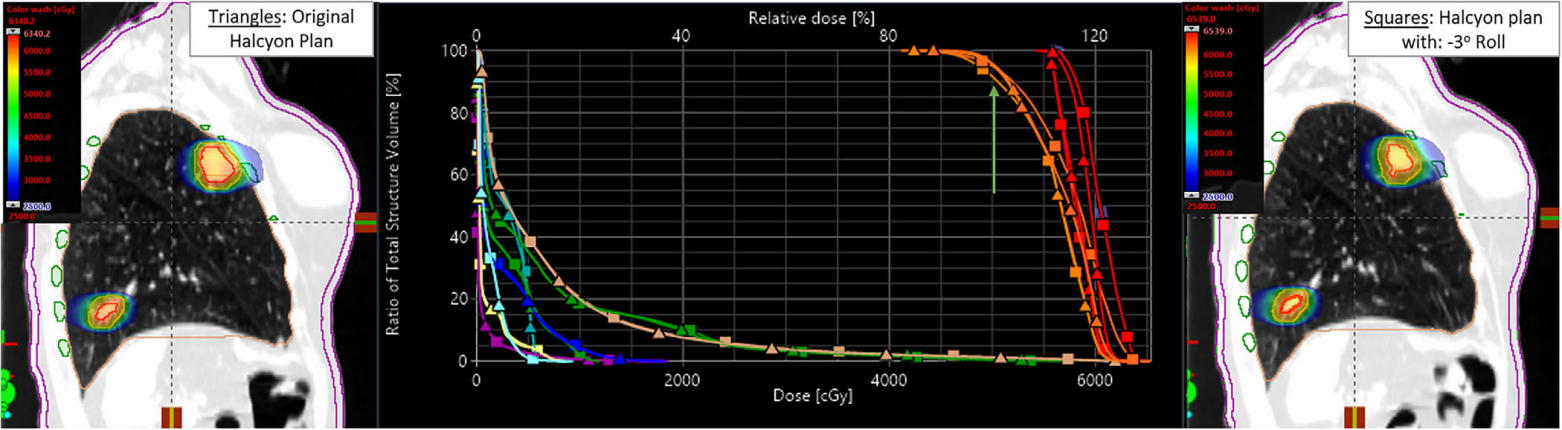
(Example uni‐lateral SIMT Lung SBRT plan on Halcyon, patient #15): Left: Original isodose colorwash in the sagittal view of uni‐lateral left lung lesions SIMT‐SBRT plan on Halcyon for 50 Gy in five fractions to each lesion, simultaneously. Middle: comparision of DVHs parameters, triangles representing the original SIMT plan and squares representing the transformation plan with −3^o^ roll applied to the original SIMT‐SBRT plan. Right: corresponding isodose colorwash distribution is shown for the example case. Crosshair shows the isocenter location. The average distance to isocenter was 6.5 cm. The vertical green arrow shows the PTV(D100%) loss of both PTV1 and PTV2 with respect to the origial PTVs coverage by 4.5% and 1.2%, respecitvely. Although, changes in mean dose to ITVs (red) due to −3^o^ roll was clinically insignificant. PCI lost was 11.6% and 4.0% for PTV1 and PTV2, respectively. However, no clinically significant increase of maximal dose to heart (blue), spinal cord (yellow), esophagus and bronchial tree was observed. However, the maximal dose to ribs (green) was lower by 2.0 Gy.

For analyzing the loss of target(s) coverage, in addition to the percentage of each PTV volume covered by the 100% isodose line as PTV(D100%), Paddick conformity index (PCI) was calculated for each target using Paddick's formula.^39^ To assess the global maximum dose of each plan, heterogeneity index (HI) was calculated as the ratio of PTV maximal point dose and prescription dose. Minimum, maximum, and mean dose to each ITV were assessed as a function of rotational patient setup errors (see Figures 2 and 3). Doses to OAR were evaluated including maximum dose to 0.03 cc of ribs, spinal cord, heart, bronchial tree, esophagus, and skin per RTOG protocols. The doses per angle and axis of rotation were averaged together and a standard deviation was calculated.

Figure 2 shows the comparison of original SIMT Halcyon SBRT plan and simulated Halcyon plan for +3^o^ roll applied to the transformed CT images (axial views), used for one of the patients with centrally located bilateral synchronous 2 lung lesions SBRT plan. There was a significant loss in the target's coverage and PCIs due to rotational patient setup errors by up to 4%. A clinic would be unable to correct for these losses with the current Halcyon design, this indicates a potential underdosing of the tumors. Moreover, due to the proximity of the critical organs for centrally located lesions, the maximum dose to OAR was higher on Halcyon, including dose to heart and spinal cord went up by 9 Gy and 2.5 Gy, respectively.

Similarly, Figure 3 demonstrates the comparison of original SIMT Halcyon SBRT plan vs simulated Halcyon plan for −3^o^ roll applied to the transformed CT images (sagittal views), used for one of the patients with peripherally located unilateral synchronous two lung lesions SBRT plan. It has been observed that there was a significant loss in the target's coverages up to 4.5% and PCIs due to rotational patient setup errors. This could lead to similar underdosing of the tumors as the previous example. Despite this, due to the remoteness of the critical organs, there was not a major concern of introducing unwanted treatment related toxicity to the patients for peripherally located lung lesions, thus maintaining maximal and volumetric dose to the OAR including normal lung dose–that was within NRG‐BR001/002 guidelines.

## 3 RESULTS

**FIGURE 4:**
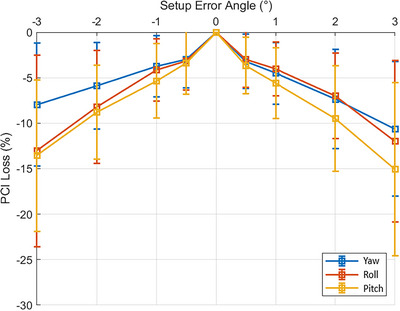
Percentage loss of Paddick's conformity index on simulated plans as a function of rotational setup errors on Halcyon with error bars demonstrating a 1‐σ deviation in each direction. In this cohort, anything greater than ±1^o^ rotational patient set up error in any direction provided that unacceptable loss of target(s) conformity with standard deviation up to −9%, and not recommended for SIMT lung SBRT treatment on Halcyon RDS.

**FIGURE 5:**
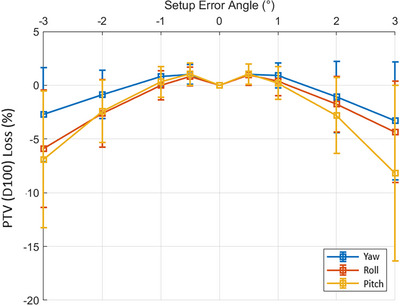
Percentage loss of PTV(D100%) target coverage on simulated plans as a function of rotational setup errors on Halcyon with error bars demonstrating a 1−*σ* deviation in each direction. In this cohort, up to ±1^o^ rotational set up error in any direction there was no noticeable change in PTV(D100%) coverage compared to the original plan and provided acceptable target coverage within ±2^o^ with and average loss of about 2.5% in the yaw and roll rotation axes, although the highest deviation was seen in the pitch rotation of up to −6%, suggesting that unacceptable loss of PTV(D100%). Thus, not recommended for SIMT lung SBRT on Halcyon RDS with more than ± 1^o^ rotational errors in this setting for oligometastatic lesions.

After simulation of rotational patient setup errors, all targets (PTVs, and ITVs) had a loss of dosimetric coverage. Compared to the original SIMT‐SBRT plans, simulated SIMT lung SBRT VMAT plans with 3° rotational errors on Halcyon demonstrated an average PTV coverage loss of 3.3 ± 5.5% (max of 8.1%), 4.4 ± 4.7% (max of 8.8%) and 8.2 ± 8.2% (max of 16.7%) for yaw, roll, and pitch rotation, respectively. Figure 4 shows the data analysis for PCI loss for all 34 lesions of 17 patients for rotational angles within [−3^o^, +3^o^] with a ± 1^o^ interval in each rotation axis, suggesting that maintaining an acceptable target coverage (average within ± 5%) within ± 1^o^, however rotational errors larger than ± 1^o^ would present serious concern for potential geometric miss via SIMT‐SBRT treatment on Halcyon RDS. Similarly, Figure 5 shows the data for loss of PTV (D100%) due to rotational patient setup errors simulated for all lesions. Throughout all metrics, the yaw axis appears to impact the dose distribution the least, with roll and pitch being slightly more severe. The drastic decrease in average PCI for the simulated SBRT plans suggests that the prescription isodose volume was not covering the planned PTV as originally intended for treatment. However, HI was relatively unaffected by rotational setup errors up to ± 3^o^, remaining effectively constant at 1.21 ± 0.04 (range, 1.15−1.31), similar to the original plans and located within the ITVs. In addition, the mean dose to ITV was 53.8 ± 1.9 Gy (range, 50.8−57.8 Gy). This is greater than nominal prescription of 50 Gy in five fractions, suggesting that with this rotational patient setup errors, ITV doses were not compromised.

**FIGURE 6:**
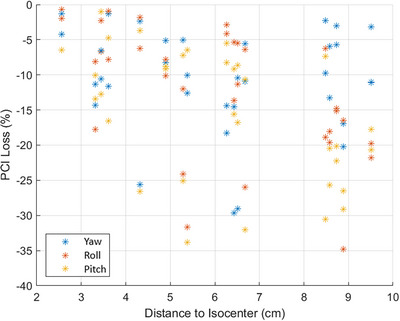
Scatter plot of relative percentage PCI loss for all 34 SIMT lung SBRT lesions as a function of distance to isocenter. For systematically assigned rotational 3° error in each direction, no clear relationship between the loss of percentage PCI and distance to isocenter was observed suggesting that there would be other variables such as irregular target sizes, tumor locations and lung heterogeneity that could have significantly impacted the variable target conformity on Halcyon for SIMT treatments.

**FIGURE 7:**
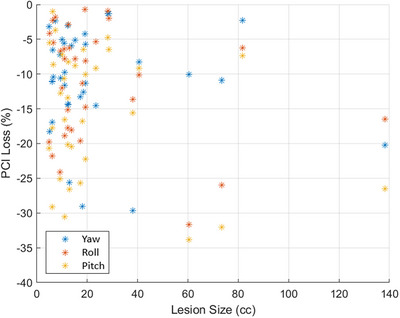
Scatter plot of relative percentage PCI loss (as shown by negative sign) for all 34 lesions as a function of tumor size with systematically simulated rotational 3^o^ error in each direction. The largest PCI lost was seen up to 32% from the original plan, however no clear relationship between the loss of percentage PCI and tumor size was seen suggesting that it will be impractical to predict actual loss of PCI in the real clinical SIMT lung SBRT cases.

When analyzing the dosimetric impact of distance to isocenter on the PCI, Figure 6 shows that the results are far from well behaved in a real‐clinical scenario of irregular target volumes of different PTV sizes including highly heterogenous dose medium for lung SBRT treatment. There is a notable trend on the expected maximum error that may be observed, but it is evidently not possible to accurately predict an estimated impact as shown in Figure 6. Given the concept of spatial localization accuracy in the SIMT setting, the expectation was to observe a downward trend of PCI loss as a function of distance to isocenter from the top left corner to the bottom right. However, in this cohort, no such clear trend was observed. Similarly, when analyzing the dosimetric impact of lesion size on the PCI, an upward trend was expected from the bottom left corner (see Figure 7). The actual results were very unpredictable with no clear trend seen, corroborating the claim that actual clinical scenarios could vary greatly from predicted values simulated with spherical targets at fixed distance, and many other factors may contribute to target dose coverage loss as discussed above.

**FIGURE 8:**
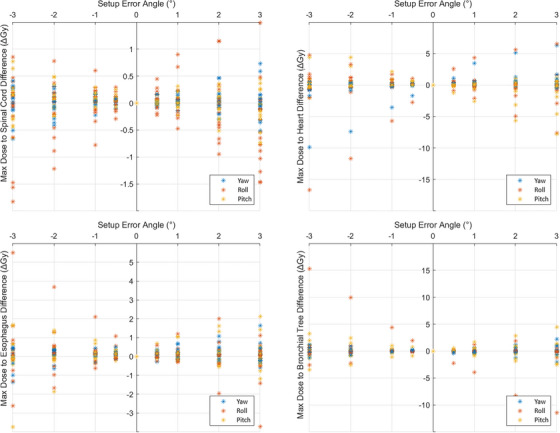
Scatter plots with all data shown for the increase (positive sign) or decrease (negative sign) of maximal dose to the spinal cord (top left), heart (top right), esophagus (bottom left), and bronchial tree (bottom right) as a function of simulated patient setup errors for all 17 SIMT lung SBRT cases. Doses vary significantly while demonstrating a bowtie‐shaped distribution, showing that exact dose differences to OAR cannot be easily predicted in real world cases, but provides some evidence of expected ranges for each angle of rotation axis.

The differences in maximal dose to OAR were also not well predictable, although still demonstrated some key patterns in this simulation study. Figure 8 shows the differences in maximal dose to four immediately adjacent vital critical organs: spinal cord, heart, esophagus, and bronchial tree. Maximum dose to the spinal cord did not change significantly between the original and simulated VMAT plans due to the fact that spinal cord was typically away from the lung lesions. However, the cohort with ±2^o^ of rotational setup error, the largest increase in maximal dose to heart was up to 5 Gy in yaw and roll direction: that would be unacceptable per RTOG‐0813/NRG‐BR001/002 protocols requirements.^1^, ^2^, ^38^ What we have observed is that the change in maximal doses falls within a distinct bowtie shaped distribution, demonstrating that exact dose differences may not be easily predictable due to the complexity of the patient's anatomy. However, the limits of typical dose differences increase with increasing rotational setup errors on Halcyon with maximum possible observed dose differences up to 5 Gy to heart, 3.8 Gy to esophagus and 10 Gy to bronchial tree in the pitch rotation with ±2° for these critical organs as demonstrated in Figure 8. Due to these rotational setup errors, clinically insignificant dosimetric variation was observed on the volumetric dose to these critical organs on Halcyon RDS (numbers are not reported here); only concern was loss of target coverage and conformity and significant change in maximal dose to OAR.

In this patient cohort, the changes in maximal dose to critical organs were relatively lower in the roll and yaw rotations, as compared to pitch direction. Moreover, as shown in Figure 8, the maximum dose to critical organs did not always increase due to rotational patient setup errors. For instance, in many cases, the maximal dose decreases from the original plans up to 1.4 Gy (spinal cord), 11 Gy (heart), 2 Gy (esophagus) and 2.5 Gy (bronchial tree) at 2^o^ rotational error in the pitch direction.

## 4 DISCUSSION

A clinically realistic and useful tool was developed to simulate and quantify the dosimetric impact of rotational setup errors for single‐isocenter two separate lung lesions SBRT plans on Halcyon RDS. After applying these clinically observable pre‐treatment conebeam CT rotational errors of up to ±3° in each direction, in this cohort, any rotational setup error greater than ±1.5^o^ in any direction provided unacceptable loss of PCI for these lung lesions with standard deviation (1−σ) up to 9% in some cases. This suggests not using SIMT setting on Halcyon under these circumstances (Figure 4). Similarly, dramatic loss of PTV(D100%) coverage was observed with a 3^o^ rotational error resulting in an average relative dose coverage loss of −3.3%, −4.4% and −8.2% for the yaw, roll, and pitch axes (up to −8.1%, −8.8% and −16.7% in some cases, Figure 5), respectively. However, in this cohort within ±2° rotational setup error the average loss of PTV(D100%) was −2.5% in the yaw and roll directions, although the most severe deviation was observed in the pitch rotation that was up to −6%, supporting the requirement to maintain rotational patient setup errors of less than ±1.5^o^ in each direction for treatment of SIMT‐SBRT on Halcyon for effectively managing oligometastatic lesions.

The process of transferring the results from simulated perfect world single‐isocenter/2‐lesion lung SBRT dosimetric impact results on Halcyon to the actual clinical environment was shown to be complicated and unpredictable. We believe that for these lung SBRT plans, the significant loss of target conformity could be due to irregular target sizes, tumor locations, steep dose gradient, and highly heterogenous lung tissues that negatively impacting the target coverage and dose conformity. Due to the complex patient's anatomy and other variables as mentioned above, there was no strong trend of predicable loss of target coverage and conformity as a function of distance‐to‐isocenter or target size as shown in Figures 6 and 7.

Due to these rotational setup errors that cannot be corrected on Halcyon, the patient anatomy could receive an excessively high dose to adjacent critical organs, even though the original SBRT plan met the NRG‐BR001 requirements, as shown in Figure 8. Therefore, caution must be taken when selecting a patient for single‐isocenter SBRT treatment given the potentially large reduction in target coverage or increase in maximal dose to adjacent OAR. There did not appear to be any single variable that dominates the PTV coverage loss (such as distance to isocenter or PTV size), implying all factors must be taken in to account when considering how robust the SIMT lung SBRT plan is and assign an appropriate margin to each lesion. As mentioned above, depending on the proximity to the target, sizes and locations, the OAR could receive substantially higher doses than anticipated with relatively little setup error on Halcyon, as reported for up to 3^o^ rotation in any direction (see, Figure 2).

For many SBRT patients, retaining treatment position on the couch for long period of time may be uncomfortable and result in intrafraction motion error, causing the desire for faster, yet effective SBRT treatments to be delivered. Many previous researchers have reported the concern of intrafraction motion errors in lung SBRT.^40^, ^41^, ^42^ For instance, Bissonnette et al.^40^ demonstrated that spatial errors, although typically small in lung SBRT, could be larger with longer treatment times for a patient on the table. Another previous study by Hoogeman et al.^41^ reported that intrafraction setup errors will increase linearly with patient treatment time on the table, providing incentive to decrease the overall treatment time and emphasizing SIMT‐VMAT planning and delivery. For 6MV‐FFF beam, the TrueBeam linac allows for a higher maximum dose rate up to 1400 MU/min while the Halcyon RDS maximum achievable dose rate of 800MU/min potentially increasing the beam‐on time. However, the slower gantry rotation speed on TrueBeam (conventionally, at least one minute per revolution) can limit the overall treatment time throughout the treatment whereas Halcyon is able to maintain delivery at the maximum dose rate of 800 MU/min at every control point and up to 4 times faster the gantry and twice the MLC rotation speed. A potential Vendor upgrade of Halcyon's maximal achievable dose rate of up to 1000 MU/min could significantly result in much faster beam‐on times similar or even faster to that of 6MV‐FF TrueBeam delivery and further reduce overall treatment times. Although this simulation study does not account for intrafraction patient setup errors, this consideration would add further uncertainty when delivering lung SBRT on Halcyon RDS. However, treating patients faster via SIMT‐SBRT could minimize intrafraction patient motion errors and improve patient comfort and compliance. Additionally, it will improve clinic workflow and patient throughput a larger patient cohort is ongoing to see the effectiveness of this treatment approach, if delivered correctly.

Moreover, a previous study by Clark et al.^43^ demonstrated the dosimetric impact of rotational patient setup errors for SIMT‐VMAT SRS to multiple brain lesions on C‐arm linac using a third‐party software. It was reported that minimizing rotational setup errors was essential for achieving adequate target coverage, even more so for small lesions in the brain and lesions far from the isocenter location. Moreover, the previous study found that even 2° rotation in any direction could reduce PTV target coverage by 89.4 ± 10.6%, up to 100% in some cases. In contrast, in our study we simulated dosimetric impact of rotational patient setup uncertainty on ring‐gantry Halcyon linac and recommended patient setup tolerances for rotational corrections that can be made. For SIMT‐SBRT treatment, we found that with 2° rotation that could reduce PTV target coverage with an average of 2.5%, up to 6% in some cases in the pitch direction. This current study does not use a third‐party software but rather a novel tool that was integrated into the Velocity software to preserve all treatment planning parameters including planning CT images, dose calculation algorithm, and structure contours in Eclipse TPS, therefore introducing no additional sources of dose calculation errors. This simulation tool that incorporates Velocity software into Eclipse TPS can be used for both extracranial as well as intracranial lesions for simulating the dosimetric impact and characterizing the potential use of SIMT‐SBRT/SRT treatments on Halcyon and any C‐arm linacs, as needed.

Despite the growing interest in SIMT lung SBRT treatments, difficulties due to daily patient setup errors on Halcyon RDS have been described here. One limitation of this study is that it is retrospective in nature. As such there are no reported clinical outcomes at this time to demonstrate the effectiveness of the two synchronous lesions lung SBRT treatment on Halcyon with these simulated dosimetric impacts. Many factors play into what clinically acceptable amount of rotational setup error can be afforded. Based on this simulation study, we recommend no more than ±1^o^ rotational tolerance in each direction for patient setup and alignment and recommend usage of rotational correction whenever possible, although there is no way to automatically determine and apply ± 1^o^ patient rotation setup error on current Halcyon Linac. However, in our clinical experience and the limited data analyzed here, the incidence of percentage of less than ±1^o^ of patient's rotational set up in each rotation axis was approximately less than 20% of the time on current TrueBeam SIMT treatments. Thus, the careful thought into OAR geometric location with respect to the target(s), sizes of the targets, distances from the isocenter, beam geometry, and patient tolerability should be considered. Given the current lack of a mechanical rotational correction on Halcyon such as 6DOF table top, it appears imperative to the expansion of Halcyon RDS based SIMT lung SBRT programs to work towards the development of a solution for accurately correcting patient setup before treatment. This could be a vendor‐developed retrofitted 6DOF couch or the development of a virtual gantry/collimator rotation correction strategy for roll that could potentially apply the concepts of adaptive radiotherapy treatment on Halcyon RDS. In this cohort, due to the anatomical complexity, there was no strong predictor of loss of target(s) coverage and conformity was not seen as a function of distance to isocenter or lesion size in the SIMT setting. Thus, the multivariable data analysis is under way to fully characterize Halcyon RDS for SBRT treatment of two lung lesions, synchronously. Furthermore, our ongoing clinical research includes simulating dosimetric impact of rotational patient setup errors for site‐specific extracranial oligometastatic SIMT‐SBRT treatment plans including double‐vertebral spine, multi‐lesion abdominal/pelvis lymph nodes, multi‐lesion liver SBRT, and performing multivariable data analysis to fully characterize the Halcyon RDS for site‐specific patient setup error tolerance for SIMT treatments in the future.

## 5 SUMMARY AND CONCLUSION

This clinically realistic simulation study demonstrates that dosimetrically it was acceptable to treat single‐isocenter/2‐lesion lung SBRT on Halcyon RDS within ±1° rotational patient setup error in any axis. However, severe dosimetric impact of significant loss of target(s) coverage, conformity, and excessively higher maximal dose to adjacent OAR was clinically observed due to greater than 1° rotation in any axis. This warrants caution when selecting, planning, and delivering SIMT lung SBRT on Halcyon RDS.

## AUTHOR CONTRIBUTIONS

Damodar Pokhrel conceptualized the project. Richard Mallory and Damodar Pokhrel performed the simulation of dosimetric impact of rotational patient set up corrections on coplanar geometry Halcyon RDS for two‐lesion lung SBRT. Richard Mallory collected and analyzed the data. Mark E Bernard and Mahesh Kudrimoti provided their clinical expertise for SIMT lung SBRT treatments, and supervision of the paper. Damodar Pokhrel, and Richard Mallory drafted the preliminary manuscript. All co‐authors revised the draft and approved the final manuscript for submission.

## CONFLICT OF INTEREST STATEMENT

The authors declare no conflict of interests.

## Data Availability

Research data are not shared.
